# Nanovesicles from adipose-derived mesenchymal stem cells inhibit T lymphocyte trafficking and ameliorate chronic experimental autoimmune encephalomyelitis

**DOI:** 10.1038/s41598-018-25676-2

**Published:** 2018-05-10

**Authors:** Alessia Farinazzo, Stefano Angiari, Ermanna Turano, Edoardo Bistaffa, Silvia Dusi, Serena Ruggieri, Roberta Bonafede, Raffaella Mariotti, Gabriela Constantin, Bruno Bonetti

**Affiliations:** 1 Department of Neurological, Biomedical and Movement Science, University of Verona, Verona, Italy; 2 Department of Medicine, Section of General Pathology, University of Verona, Verona, Italy; 3 Neurology Unit, Azienda Ospedaliera Universitaria Integrata Verona, Verona, Italy

## Abstract

Cell based-therapies represent promising strategies for the treatment of neurological diseases. We have previously shown that adipose stem cells (ASC) ameliorate chronic experimental autoimmune encephalomyelitis (EAE). Recent evidence indicates that most ASC paracrine effects are mediated by extracellular vesicles, i.e. micro- and nanovesicles (MVs and NVs). We show that preventive intravenous administration of NVs isolated from ASC (ASC-NVs) before disease onset significantly reduces the severity of EAE and decreases spinal cord inflammation and demyelination, whereas therapeutic treatment with ASC-NVs does not ameliorate established EAE. This treatment marginally inhibits antigen-specific T cell activation, while reducing microglial activation and demyelination in the spinal cord. Importantly, ASC-NVs inhibited integrin-dependent adhesion of encephalitogenic T cells *in vitro*, with no effect on adhesion molecule expression. In addition, intravital microscopy showed that encephalitogenic T cells treated with ASC NVs display a significantly reduced rolling and firm adhesion in inflamed spinal cord vessels compared to untreated cells. Our results show that ASC-NVs ameliorate EAE pathogenesis mainly by inhibiting T cell extravasation in the inflamed CNS, suggesting that NVs may represent a novel therapeutic approach in neuro-inflammatory diseases, enabling the safe administration of ASC effector factors.

## Introduction

Multiple sclerosis (MS) and its animal model experimental autoimmune encephalomyelitis (EAE) are immune-mediated disorders of the central nervous system (CNS), characterized by inflammation, demyelination and axonal degeneration^1,2^. Pre-clinical data obtained in EAE models show that adult stem cell-based therapies contribute to the prevention and/or repair of the CNS damage by a dual mechanism: modulating the immune system when administered in the pre-clinical phase and promoting remyelination through the activation of oligodendrocyte precursors when injected in the chronic phase of disease^3–11^. Among stem cell sources, mesenchymal stem cells (MSC) are stromal progenitor cells derived from different tissues that represent a promising therapeutic tool for autoimmune inflammatory diseases like MS, due to their immuno-modulatory effect and neuroprotective ability^7^. Adipose-derived MSC (ASC) can be easily isolated from adipose tissue and share morphology, immune phenotype, rate of isolation and paracrine behavior with bone marrow-derived MSC^12,13^. ASC limit EAE induction and ameliorate chronic EAE when administered after disease stabilization, leading to the reduction of CNS inflammation and demyelination and induction of local neuroregeneration^11^.

Stem cells (SC) injected systemically can stably integrate for prolonged time periods in target and non-target organs with unpredictable consequences, and a number of concerns have been raised in relation to the potential side effects of SC-based therapies. Among these, several studies have shown that tumor growth depends on the interaction with stromal cells and MSC may promote tumor cell growth by modulating tumor micro-environment^14,15^. In addition, SC injected at high concentration into tissues form aggregates and may generate their own microenvironment, leading to the formation of bone nodules or other undesirable structures. Furthermore, MSC rapidly undergo cell-to-cell adhesion with the risk of pulmonary emboli or infarctions if not carefully prepared for intravenous infusion^16^.

A growing body of literature indicates that ASC exert their action by producing soluble factors, which modify the microenvironment resulting in neuroprotective and immuno-modulating effects^17–19^, and this paracrine effect provide the basis for an attractive non-cell-based therapy for autoimmune disorders. Among these factors, two types of extracellular vesicles have been recently studied: nanovesicles (NVs) and the larger microvesicles (0.1–1 µm)^20,21^. NVs are 40–100 nm diameter vesicles that are secreted upon fusion of multivesicular endosomes with the plasma membrane, serving as information packets, which can be transferred to other cells and modulate their functions^22,23^. The protein composition of NVs varies depending on the cell type of origin and unique tissue/cell type signatures have been identified for NVs. However, in spite of their cellular origin, a conserved set of proteins are common to NVs, including ALG-2-interacting protein X (Alix), Tumor susceptibility gene 101 (TSG101), CD9, CD81 and heat-shock protein 70 (HSP70)^24^.

Here we show that the preventive administration of NVs isolated from ASC (ASC-NVs) ameliorates chronic EAE by inhibiting T cell adhesion in inflamed CNS venules and trafficking to the CNS in EAE mice, leading to reduced spinal cord inflammation, microglial activation and demyelination. Differently from their parental cells, ASC-NVs had no effect when administered at disease onset and displayed limited effect on T cell activation and cytokine production. Our data suggest that ASC-NVs may represent a novel therapeutic approach for neurological autoimmune diseases, including MS.

## Results

### Preventive administration of ASC-NVs reduces clinical severity and neuropathological changes in chronic EAE

**Figure 1:**
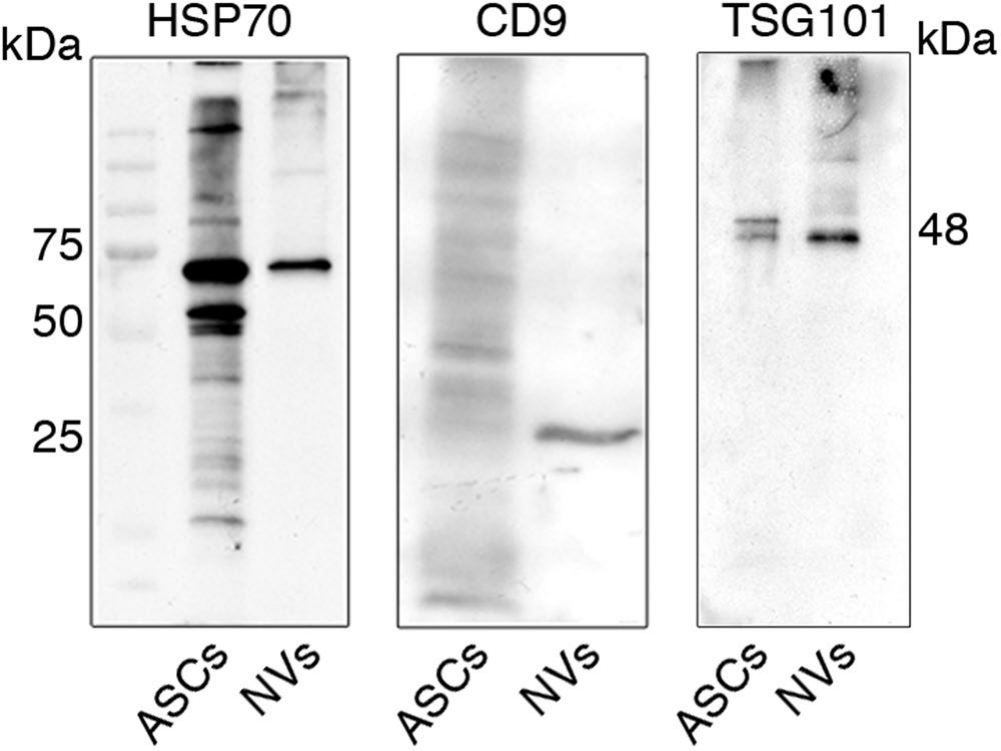
Characterization of ASC-NVs by western blot analysis. Protein lysates from ASC cells and ASC-NV fractions were separated on polyacrylamide gels and probed with specific NVs markers. In NV fractions, a single band at 70 kDa and around to 25 kDa was present after incubation with HSP70 and CD9 antibodies, respectively. Similarly, the treatment with anti-TSG101 antibody displayed an intense signal at 48 kDa in NV populations. Moreover, ASC cells expressed both HSP70 and TSG101 markers whereas showed weak reactivity against CD9 antibody.

**Figure 2:**
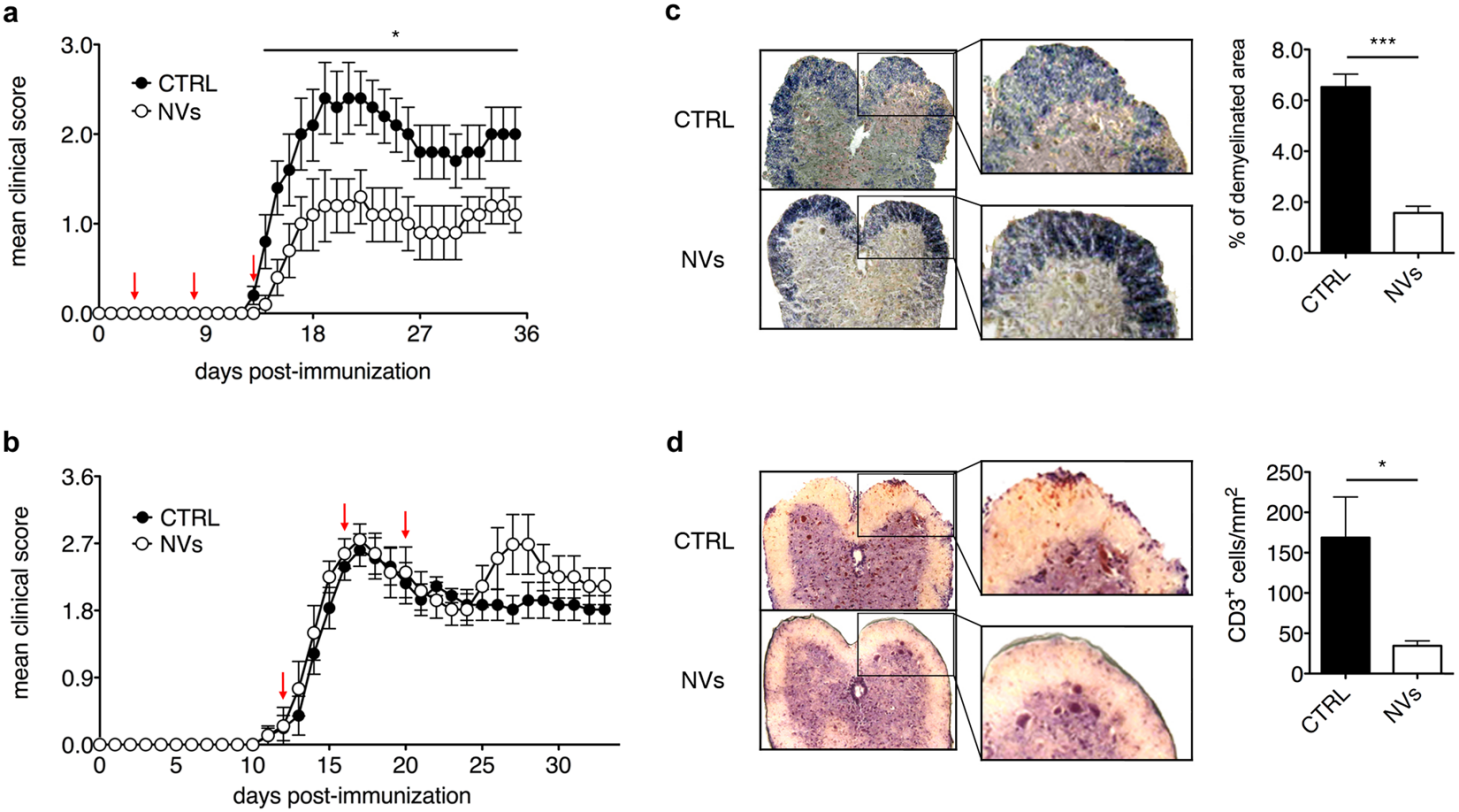
Preventive administration of ASC-NVs ameliorates EAE course. (**a**) MOG_35–55_-immunized C57Bl/6 mice were treated with PBS (CTRL condition) or 5 μg ASC-NVs at 3, 8 and 13 dpi (red arrows). ASC-NVs significantly inhibited the mean clinical score and chronic EAE development, compared to CTRL condition (day 14 p = 0.041; day 15 p = 0.019; day 16 p = 0.048; day 17 p = 0.045; day 18 p = 0.046; day 19 p = 0.044; day 20 p = 0.033; day 21 p = 0.021; day 22 p = 0.019; day 23 p = 0.020; day 24 p = 0.030; day 25 p = 0.018; day 26 p = 0.018; day 27 p = 0.018; day 28 p = 0.030; day 29 p = 0.049; day 30 p = 0.048; day 31 p = 0.048; day 32 p = 0.041; day 33 p = 0.032; day 34 p = 0.032; day 35 p = 0.024). Data are the mean ± SEM of three independent experiments, for a total of 15 mice/condition. Results are also summarized in Table 1. (**b**) MOG_35–55_-immunized C57Bl/6 mice were treated with PBS (CTRL condition) or 5 μg ASC-NVs at 12, 16 and 20 dpi (red arrows). ASC-NVs did not impact EAE development when injected after disease onset. Data are the mean ± SEM of 9 mice/condition. (**c**) Woelcke staining of lumbar spinal cords showed a reduction of demyelination in mice treated with ASC-NVs, compared to control mice (p = 6.19E-06). Such beneficial effect by ASC-NVs is markedly visible in the blow-up. See also Table 1 for quantification. (**d**) Immunohistochemistry for CD3^+^ T lymphocytes on serial sections showed a decreased number of infiltrating CD3^+^ T cells in EAE lesions from mice treated with ASC-NVs, compared to controls (p = 0.02), as evident in the inserts. See also Table 1 for quantification. For (**c**) and (**d**), the data are the mean ± SEM of three independent experiments.

Our previous studies have shown that systemic treatment with ASC during the preclinical phase of disease prevents chronic EAE development^11^. To assess the effect of ASC-NVs on EAE, we produced NVs from cultured ASC and characterized them by western blot (Fig. 1). Mice immunized with MOG_35–55_ peptide were treated with PBS (control animals) or NVs before the clinical signs of disease (Fig. 2a) or at disease onset (Fig. 2b). Control mice developed the first clinical signs of EAE at 16.3 ± 1.2 days post-immunization (dpi) (mean ± SEM), reached a peak around 20 dpi and then presented a stable disease course, typical of this chronic EAE model (Fig. 2a and Table 1). Notably, differently from their parental ASC, treatment with ASC-NVs after disease onset failed to modify the clinical course of EAE (Fig. 2b). As expected, the pathological analysis of spinal cord sections in all control mice showed the presence of demyelinated areas and inflammatory infiltrates (Fig. 2c,d). Notably, mice treated with ASC-NVs before disease onset showed a drastic reduction of the mean clinical score (Fig. 2a), maximum clinical score and cumulative score (Table 1), compared to control animals. In addition, neuropathological studies showed a significant reduction of the areas of demyelination and in the number of CD3^+^ T cells infiltrating the CNS at disease peak (Fig. 2c,d; Table 1). Taken together, these results demonstrate that ASC-NVs prevent chronic EAE development by limiting immune cell infiltration and CNS inflammation, whereas had no effect once inflammatory cells have already entered the CNS.

**Table 1:** Clinical and pathological features of NV-treated EAE mice.

	Disease onset	Mean maximum score	Mean cumulative score	Demyelinated area^a,b^	CD3^+^ cells/mm^2 a^
Control (16 mice)	16.3 ± 1.2	3.1 ± 0.4	42.8 ± 6.4	6.5 ± 0.5	168.7 ± 50.4
ASC-NVs (15 mice)	18.2 ± 1.4	1.7 ± 0.3^*^ p = 0.01	21.8 ± 5.3^**^ p = 0.002	1.6 ± 0.3^***^ p = 0.0002	34.4 ± 6.2^****^ p = 0.00004

The table report the data concerning disease onset, mean maximum/cumulative score, demyelinated area of spinal cord and the quantification of CD3+ cells of NV-treated EAE mice compared to control mice. ^a^Mice sacrificed at disease peak. ^b^% of total SC area.

### ASC-NVs inhibit microglia activation *in vitro* and *in vivo*

**Figure 3:**
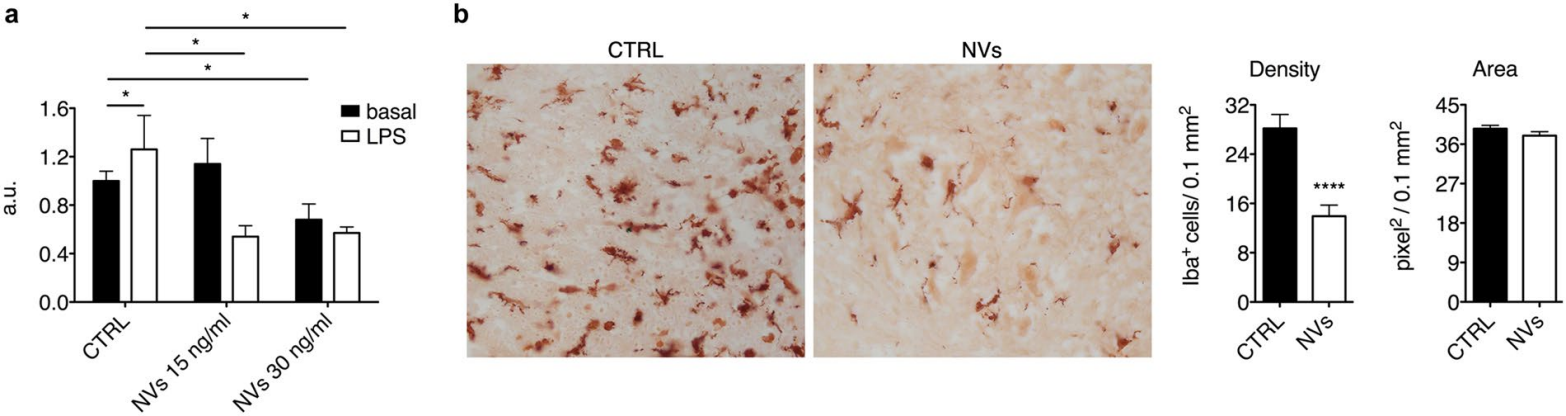
ASC-NVs inhibit microglial cell activation *in vitro and in vivo*. (**a**) N9 cells were incubated with LPS 100 ng/ml for 24 h and then treated with 15 ng/ml and 30 ng/ml of ASC-NVs for 48 h. ASC-NVs significantly inhibited microglial cell proliferation *in vitro* (cntrl basal vs cntrl LPS p = 0.035; cntrl basal vs NVs30 p = 0.039; cntrl LPS vs NVs15 p = 0.020; cntrl LPS vs NVs30 p = 0.012). Data are presented as fluorescence arbitrary units (a.u.) relative to the basal condition and are mean ± SD of a representative experiment performed in triplicate. (**b**) Evaluation of microglial activation in the spinal cord spinal cord of PBS (CTRL) or NV-treated EAE mice at disease peak. Activated microglial cells were identified by immunohistochemistry, following staining with anti-Iba-1 antibody. Treatment with NVs strongly inhibited microglial activation in EAE mice, as evident by the reduced number of Iba-1^+^ cells in the spinal cord of NV-treated animals (p = 8.11E-06). Data are the mean ± SEM of three independent experiments.

To investigate the underlying mechanisms responsible for the beneficial effect observed after the treatment with ASC-NVs, we firstly assessed the effect of ASC-NVs on microglial cells. We performed *in vitro* assays to analyze the impact of ASC-NVs on the N9 murine microglial cell line. Treatment of N9 cells with ASC-NVs significantly inhibited their basal and LPS-induced proliferation (Fig. 3a). Concerning *in vivo* experiment, we analyzed the impact of NVs treatment on microglia activation on EAE mice. In accordance with *in vitro* results, we found that the number of Iba-1^+^ cells was significantly reduced in the spinal cord of NV-treated animals, compared to CTRL mice (Fig. 3b), confirming that ASC-NVs may inhibit the activation of microglial cells both *in vitro* and *in vivo*.

### ASC-NVs partially reduce CD4^+^ T lymphocyte activation *in vitro* but not *in vivo*

**Figure 4:**
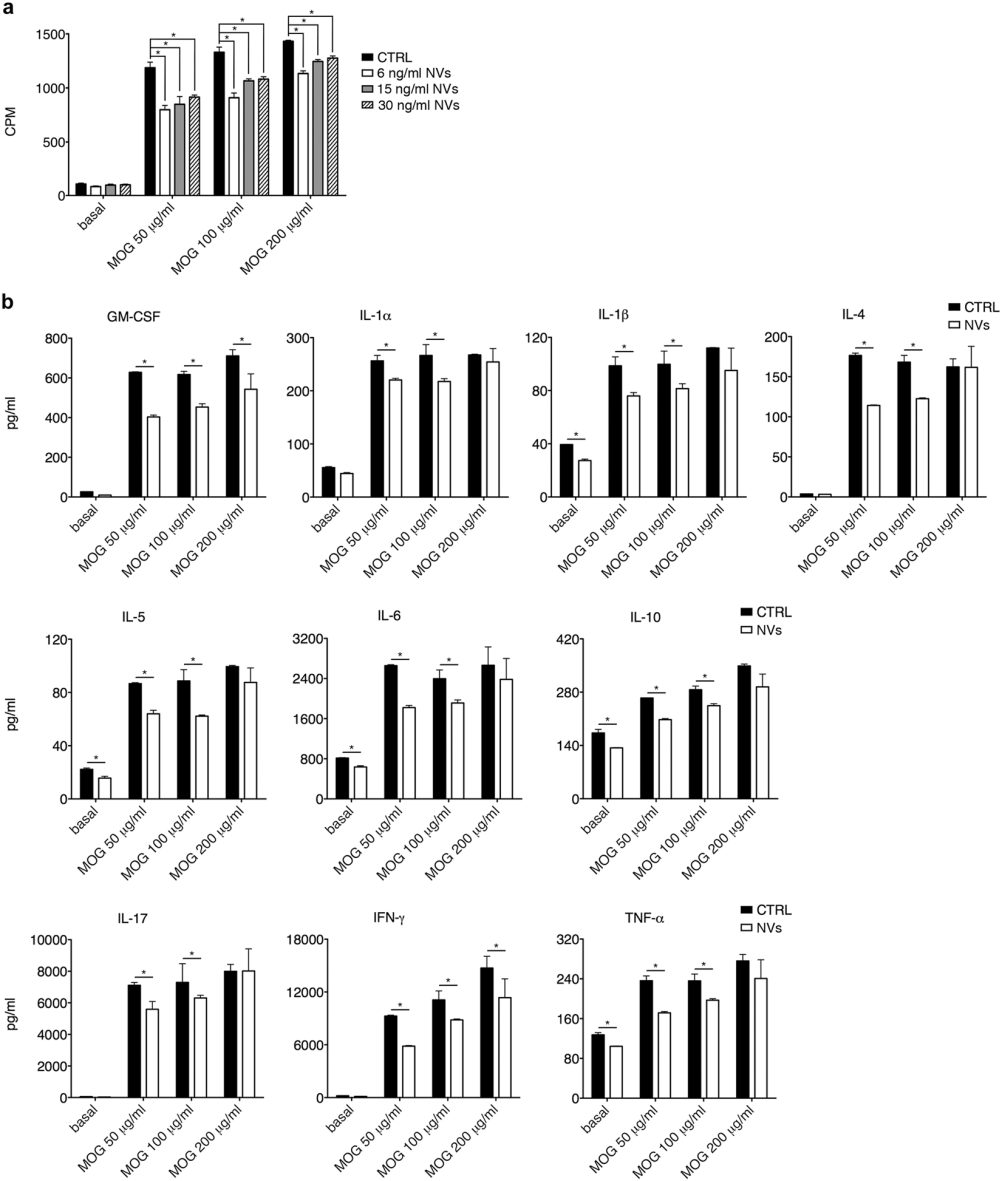
ASC-NVs partially inhibit MOG_35–55_-specific CD4^+^ T cell proliferation and cytokine production *in vitro*. (**a**) CD4^+^ cells isolated from lymph nodes and spleens of 2D2-TCR mice were re-stimulated *in vitro* for 3 days with increasing concentrations of MOG_35–55_ peptide, in the presence of irradiated antigen-presenting cells and PBS (CTRL condition) or 30, 15 or 6 ng/ml of ASC-NVs. Cell proliferation was assessed by [^3^H]-thymidine incorporation and expressed as counts per minute (CPM). ASC-NVs partially reduced antigen-specific T cell proliferation in a dose-dependent manner, when compared with control cells (*p < 0.05). Data are the mean ± SEM of three independent experiments performed in triplicate. (**b**) Secretion of cytokines (pg/ml) in supernatants by proliferating CD4^+^ T cells was also significantly affected by ASC-NVs, compared to the control condition (*p < 0.05). Data are the mean ± SD of one representative experiment from a series of two with similar results.

We next analyzed the immuno-modulatory activity of ASC-NVs on T cells. Proliferation assays performed *in vitro* showed that ASC-NVs partially inhibited antigen-specific T cell proliferation, reaching a maximum of 30% reduction (Fig. 4a). This effect was accompanied by global reduction of cytokine production by proliferating T cells, as assessed by Multiplex assay. The presence of ASC-NVs in cell cultures reduced both pro- (i.e. IL-1β, IL-1α, IL-6, IL-17, IFN-γ, GM-CSF and TNF-α) and anti-inflammatory (IL-10, IL-4 and IL-5) cytokine secretion by T cells (Fig. 4b), suggesting that ASC-NVs partially limit T cell activation *in vitro*.

**Figure 5:**
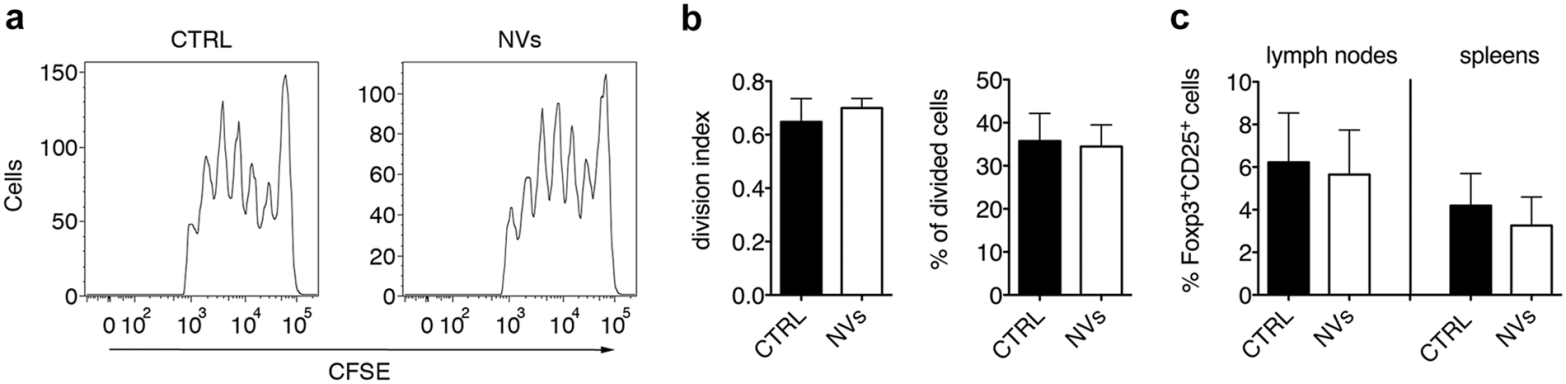
ASC-NVs do not impact CD4^+^ T cell activation *in vivo* in EAE mice. 15 × 10^6^-CFSE labeled lymph node and spleen cells from 2D2 mice were injected 8 dpi in EAE recipient mice previously treated with two PBS (CTRL) or ASC-NV injections at 3 and 8 dpi. (**a**) Representative plots from one control and one NV-treated mouse showing the proliferation of exogenous CD4^+^CFSE^+^ T cells detected as CFSE dilution from the original T cell population. (**b**) Samples were analyzed with FlowJo software to quantitatively assess T cell proliferation in recipient mice. No differences were observed between the proliferation of exogenous CD4^+^ T cells in control or NV-treated animals. Data are the mean ± SD of five mice/condition. (**c**) Quantification of Foxp3^+^CD25^+^ regulatory T cells (Tregs) in draining lymph nodes and spleens of EAE mice. Lymph nodes and spleens were collected at disease peak from EAE mice treated with PBS (CTRL) or ASC-NVs at day +3, +8 and +13 post-immunization (preventive treatment). Treatment with NVs did not impact the amount of Tregs in both lymph nodes and spleens. Data are shown as % of Foxp3^+^CD25^+^ Tregs on the total CD3^+^CD4^+^ T cell population and are the mean ± SD of 4 mice/condition.

Based on *in vitro* results, we sought to determine if ASC-NVs limited T cell activation also *in vivo* in EAE mice. To this purpose, we injected EAE mice treated or not with ASC-NVs with CFSE-labeled cells from lymph nodes and spleens of naïve 2D2 TCR-transgenic mice, which display a TCR specific for MOG peptide on their T lymphocytes. Cells were injected at 8 dpi in EAE recipient mice, which already received two systemic injections of NVs. Three days later, we evaluated the proliferation of CD4^+^ CFSE^+^ T cells in recipient mice by flow cytometry. We found that exogenous T cells efficiently proliferated in NV-treated mice, and their proliferation rate was comparable to those observed in control animals (Fig. 5a,b). These results suggest that ASC-NVs display a limited influence on T cell activation *in vivo*. Importantly, treatment with ASC-NVs also did not affect the percentage of Foxp3^+^CD25^+^ regulatory T cells (Tregs) in both lymph nodes and spleens of EAE mice (Fig. 5c), confirming lack of immune regulation by ASC-NVs *in vivo*.

### ASC-NVs inhibit integrin-dependent chemokine-induced T cell adhesion *in vitro*

The observation that ASC-NVs significantly reduce the number of T cells infiltrating EAE lesions prompted us to investigate their effect on the adhesion machinery of activated T lymphocytes. We first checked whether the administration of ASC-NVs affected the expression of adhesion molecules in EAE lesions. Immunohistochemical analysis performed on spinal cord slices from control and NV-treated animals at disease onset and peak showed no significant differences in the expression of ICAM-1, VCAM-1, P-selectin and E-selectin, suggesting that NVs do not impact endothelial activation during EAE (data not shown).

**Figure 6:**
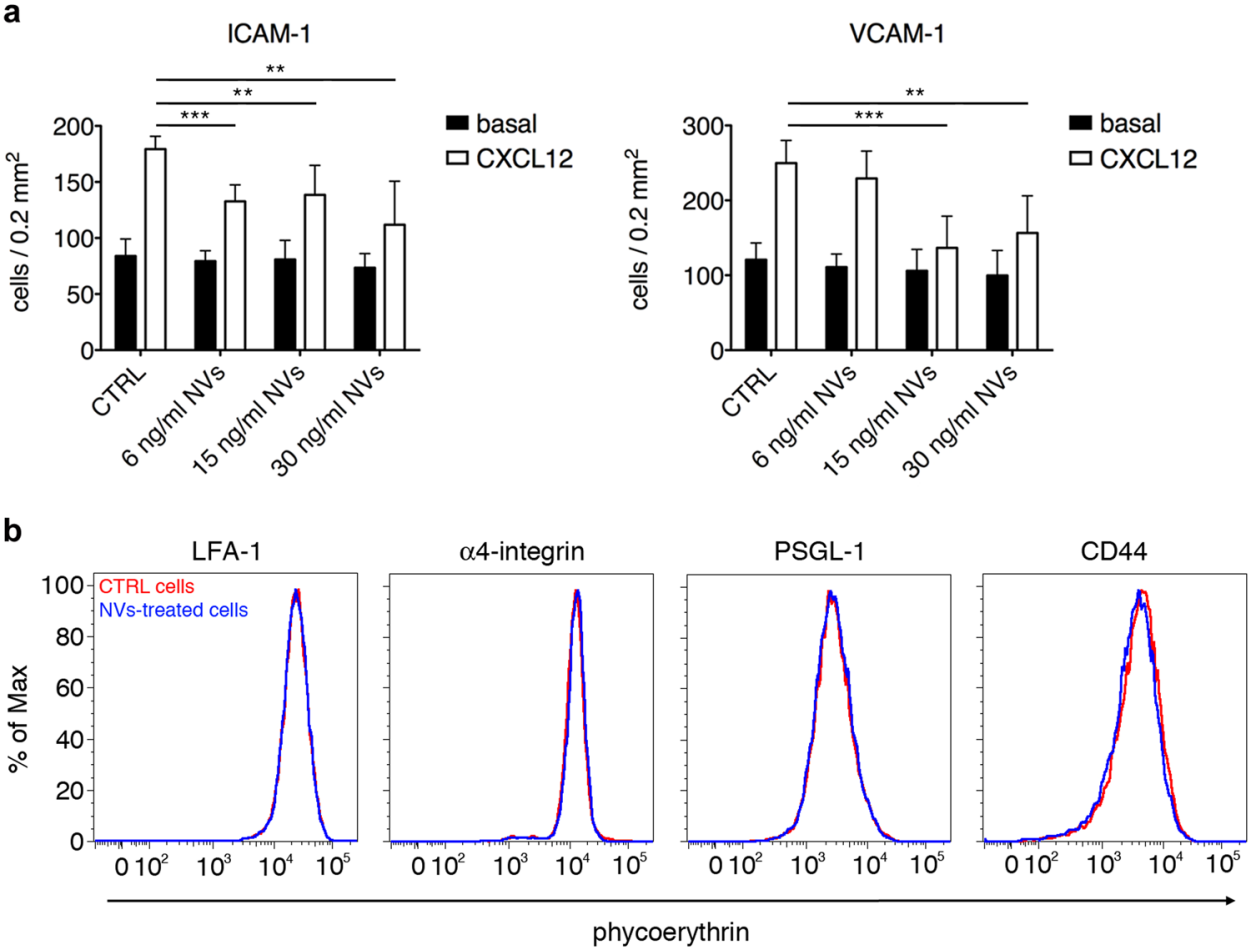
ASC-NVs inhibit activated T cell integrin-dependent adhesion *in vitro*. (**a**) MOG_35–55_-activated splenocytes from 2D2-TCR were treated for 24 hours with PBS (CTRL condition) or increasing doses of ASC-NVs. Cells were then left spontaneously adhere on purified ICAM-1 or VCAM-1 for 20 min. In some experiments, CXCL12 0.5 μM was added for 5 min to analyze chemokine-induced adhesion. Pre-treatment with NVs significantly inhibited the integrin-dependent adhesion of activated T cells induced by chemokine CXCL12, compared to control condition (ICAM-1: cntrl CXCL12 vs NVs6 CXCL12 p = 0.00011; cntrl CXCL12 vs NVs15 CXCL12 p = 0.0049; cntrl CXCL12 vs NVs30 CXCL12 p = 0.0022. VCAM-1: cntrl CXCL12 vs NVs15 CXCL12 p = 0.00054; cntrl CXCL12 vs NVs30 CXCL12 p = 0.0061). Data are the mean ± SD of three independent experiments performed in triplicate. (**b**) Adhesion molecules expression on MOG-specific activated T cells after treatment with PBS (CTRL cells) of 30 ng/ml ASC-NVs. Red line: CTRL cells. Blue line: NV-treated cells.

We next tested the direct effect of ASC-NVs on activated T cell adhesion on purified integrin ligands. Activated T cells were obtained by stimulation with MOG_35–55_ peptide of splenocytes from 2D2-TCR transgenic mice. We observed that 24 hours pre-incubation with ASC-NVs did not significantly affect the spontaneous T cell adhesion on both ICAM-1 and VCAM-1 (Fig. 6a). However, treatment of T lymphocytes with 15 or 30 ng/ml ASC-NVs strongly inhibited rapid adhesion triggered by CXCL12 chemokine (Fig. 6a). To note, treatment with NVs did not reduce adhesion molecule expression by activated T cells (Fig. 6b), suggesting that ASC-NVs selectively interfere with the signaling machinery required for integrin activation and high integrin affinity state induced by chemokines in activated T lymphocytes.

### ASC-NVs inhibit activated T cell adhesion in spinal cord venules in EAE mice

**Figure 7:**
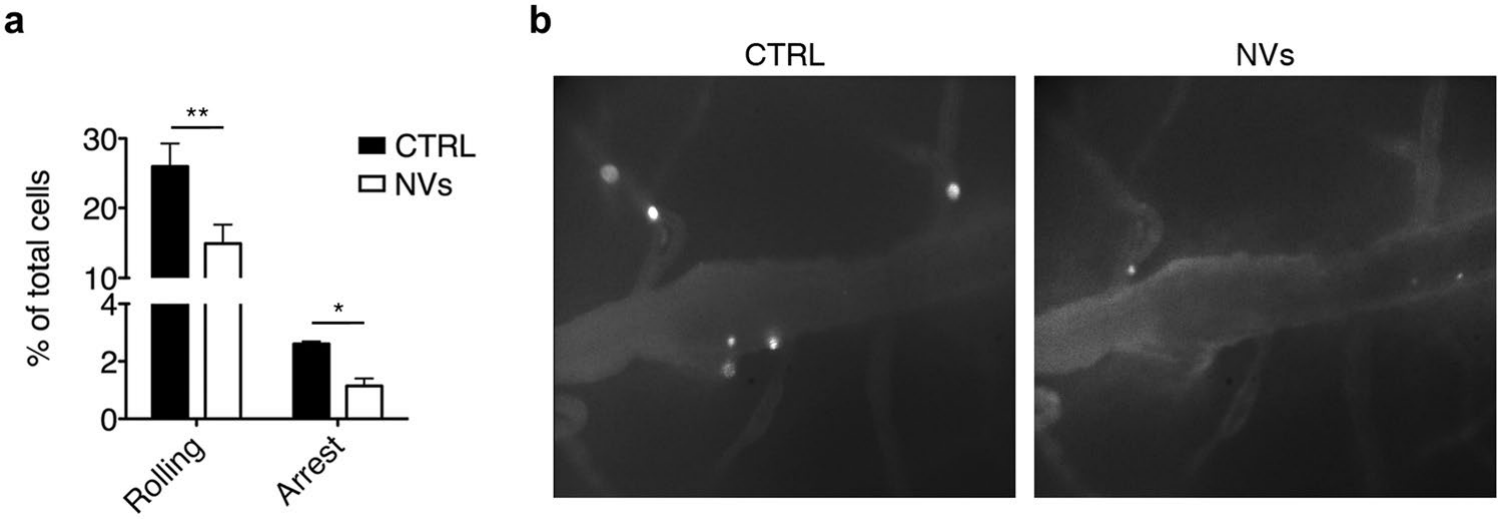
ASC-NVs block activated T cell adhesion *in vivo* in inflamed spinal cord pial vessels in intravital microscopy experiments. MOG_35–55_-activated splenocytes from 2D2-TCR were treated for 24 hours with PBS (CTRL condition) or 30 ng/ml NVs. Cells were then fluorescently labeled and injected i.v. in EAE recipient mice at disease peak. (**a**) Pre-treatment with ASC-NVs strongly reduced activated T cell adhesion in spinal cord venules, compared to control cells (rolling p = 0.0089; arrest p = 0.0325). Data are from five independent experiments, for a total of 28 venules/condition. (**b**) Representative images of spinal cord pial venules acquired during intravital microscopy experiments. Note the reduced number of adhered NV-treated cells, compared to control cells. Cells are the white spots inside venules.

The results obtained in *in vitro* adhesion assays (Fig. 6), together with the immunohistochemical analysis in mice treated with NVs (Fig. 2), suggest that ASC-NVs reduce immune cell trafficking to the CNS of EAE mice. To confirm that NVs impact activated T cell adhesion *in vivo*, we performed intravital microscopy experiments in the inflamed spinal cord of EAE mice at disease peak. To this purpose, MOG_35–55_-specific encephalitogenic T cells were treated *in vitro* with PBS (control cells) or 30 ng/ml ASC-NVs for 24 hours, and then tested for their adhesion capacity in intravital microscopy experiments performed on exposed spinal cord. We found that control cells efficiently interacted with endothelium in spinal cord pial venules at disease peak, with the majority of interacting cells performing rolling on the vascular bed, while a lower percentage of T cells firmly adhered (Fig. 7a,b). Notably, pre-treatment of MOG-specific T cells with ASC-NVs strongly reduced their ability to roll and firmly adhere in spinal cord vessels (Fig. 7a,b). These data confirm the results from *in vitro* adhesion assays and neuropathological studies, suggesting that ASC-NVs inhibit EAE development in part by blocking immune cell adhesion in activated spinal cord pial venules during disease development.

## Discussion

Stem cells are a promising approach for the treatment of autoimmune diseases of the CNS due to their immuno-modulatory effects and neuroregenerative potential, thus offering the opportunity to treat different pathological aspects of pathologies like MS. However, a major concern regarding cell-based therapies is related to the fate of systemically injected MSC, which have been demonstrated to stably integrate for prolonged time periods in target and non-target organs, with unpredictable consequences.

Growing consensus supports the idea that most of biological effects mediated by stem cells are due to paracrine mechanisms^17–19,25^. In this regard, it has been recently shown that the administration of conditioned medium from MSC may mimic the beneficial effects of MSC administration, indicating that MSC engraftment is not necessary to impact the disease. In fact, Camussi and colleagues have demonstrated that the administration of MVs derived from MSC ameliorated renal functions and improved the survival in an animal model of acute tubular injury, especially after multiple administrations^26,27^. Furthermore, it has been shown that conditioned medium from MSC reduced the infarct size and improved systolic and diastolic cardiac performance in a mouse model of myocardial ischemic injury and the beneficial effect was mediated by soluble factors contained within the 50- to 100-nm vesicles fraction^20,21^.

In the present paper, we provide evidence that ASC-NVs represent a safe and efficient approach to treat chronic EAE, avoiding the administration of parental cells. Such strategy could represent an easily transferable treatment to humans, enabling safe administration by systemic injection. In support of a potential application of NVs in humans with CNS diseases, we have recently shown that NVs (as well as MVs) are able to exert neuroprotective and neuroregenerative effects *in vitro*^28,29^. We reported that ASC-NVs protected neuronal cultures from apoptosis after oxidative stress and stimulated the process of remyelination in slice cultures after experimental demyelination with lysophosphatidylcholine.

Here we show that ASC-NVs have a significant beneficial effect *in vivo* when administered in the pre-clinical phase of chronic EAE, with amelioration of clinical score and strong reduction of spinal cord inflammation and demyelination, whereas they failed to induce any significant change in the clinical course of EAE when injected after the disease onset. In our experiments with NVs we tried to mimic the experimental setting previously employed with ASC treatment in the EAE model, performing multiple i.v. injections in the pre-clinical phase and at the disease onset with NVs obtained from approximately the same number of ASC used to treat the EAE mice. Interestingly, the comparison of the results indicates that different mechanisms are probably responsible for the beneficial effects exerted by the two treatments. In fact, the injection of ASC (and in general MSC) before the clinical onset induced a strong dose-dependent inhibition of antigen-specific T cells proliferation and a Th1-to-Th2 shift in their phenotype^7,10,11^. The effect displayed by ASC-NVs on T cell activation was marginal with a slight reduction of T cell proliferation *in vitro*; we speculate that the lack of effect on T cell activation *in vivo* reflects the fact that ASC-NVs act simultaneously on different immune cell subsets, which exert opposite effects on T cell proliferation. To note, a similar difference on proliferation between MSC and their NVs has been reported in monocytes: MSC were highly anti-proliferative, whereas NVs had a lower effect^30^. The effect on cytokine production was also significantly different as compared with ASC, since NVs induced a global down-regulation of cytokines (both pro- and anti-inflammatory molecules) without any evidence of specific effect on Th1, Th17 or Tregs sub-populations. We speculate that the effect of ASC on T cell proliferation and cytokine production is in part mediated by paracrine mechanism but mostly depends on cell-cell contacts between ASC/MSC and T cells. In addition to T lymphocytes, we focused our attention on microglial cells as possible target of ASC-NVs. Intriguingly, ASC-NVs directly induced a down-regulation of proliferation and production of pro-inflammatory cytokines by N9 microglial cell line *in vitro*, confirming a direct impact of ASC-NVs on microglial cell activation. Accordingly, treatment with NVs was associated with a significant reduction of activated microglial cells in CNS parenchyma of EAE mice. However, we cannot exclude that the reduced microglial activation observed in EAE lesions *in vivo* might be due to a lower degree of inflammation in animals treated with ASC-NVs, compared to control mice.

While the injection of ASC and ASC-NVs before the clinical onset induce similar clinical and neuropathological effects with strong reduction of infiltrating T lymphocytes and inflammatory lesions, NVs were less efficient in inhibiting autoreactive T cell activation. This discrepancy prompted us to evaluate alternative mechanisms responsible for the decreased T cell extravasation in inflamed CNS, focusing on leukocyte-endothelial interaction. Since we did not observe significant differences in endothelial expression of adhesion molecules following NV treatment in EAE mice, we focused our attention on the modulation of the adhesive capacity of activated T cells by ASC-NVs. Noteworthy, we found that the treatment with ASC-NVs inhibited the chemokine-induced adhesion of activated T cells to integrin ligands such as ICAM-1 and VCAM-1 *in vitro*, but had no effect on adhesion molecule expression, suggesting that NVs interfere with signal transduction pathways controlling integrin activation. In order to bind their ligands, integrins need to be activated through signal transduction pathways triggered by engagement of chemoattractant receptors, leading to an increased affinity and/or valency^31^. Our results suggest that NVs may inhibit integrin affinity triggering in encephalitogenic T cells. We also evaluated the effect of NVs on the adhesion of T cells in inflamed spinal cord pial venules in intravital microscopy studies^32^. We observed that encephalitogenic T cells treated with ASC-NVs displayed a significant reduction of firm adhesion in inflamed CNS vessels, compared to control cells. These data are in agreement with the reduced leukocyte infiltration observed in immunohistochemical analysis and demonstrate an inhibitory effect of NVs on T cell adhesion *in vivo*. To our knowledge, this is a previously unknown mechanism of action of MSC-derived NVs, which may help to explain the significant reduction of infiltrating T cells in EAE lesions in the absence of a strong anti-proliferative effect.

We finally compared the therapeutic efficacy of ASC-NVs injected at the disease onset. Differently from their parental cells, we observed no modification of the clinical course in this experimental setting. This result is not surprising on the basis of the previously mentioned anti-adhesive, but not cytostatic effect of ASC-NVs on autoreactive T cells, as, in the therapeutic protocol, ASC-NVs have been administered after disease onset (i.e. once inflammatory cells already entered the CNS).

In conclusion, our results show that ASC-NVs administered during early disease phases induce a beneficial effect in chronic EAE through the inhibition of trafficking of activated T lymphocytes in the inflamed CNS. Therefore, we suggest that ASC-NVs may represent a valuable tool for stem cell-based therapy in chronic inflammatory diseases of the CNS. However, it is crucial to understand the mechanisms of action of NVs and their homing after their administration. To this purpose, the use of labeled NVs with nanoparticles that allow their detection by a non-invasive technique, as magnetic resonance imaging, could be useful^33^. Once defined the mechanism(s) of action of ASC-NVs, new therapeutic approaches based on the synergistic effects of NVs with other anti-inflammatory molecules (e.g. GM-CSF as Tregs activator^34,35^) tolerance-mediated treatments through AC injection^36,37^ or drugs with complementary effects (e.g. cytostatic drugs) may be developed.

## Methods

### Cell Cultures

Murine ASC were obtained from 6–8 weeks old C57Bl/6J mice (Charles River Laboratories). The isolation of stromal-vascular fraction from adipose tissue was carried out as previously described^27,38^. Briefly, extracellular matrix was digested with Collagenase A (Roche) and centrifuged at 1200 g. The pellet obtained was re-suspended and the stromal fraction was collected by sequential centrifugation and filtration steps. Cells were then cultured in complete medium (DMEM + glucose + GlutaMAX ITM + 15% heat-inactivated adult bovine serum + penicillin/streptomycin; all from Invitrogen) supplemented with 50 ng/ml HB-EGF (R&D Systems). The immune phenotype of murine ASC was characterized by using monoclonal antibodies (mAb) specific for CD106, CD9, CD44, CD80, CD138, and Stem cells antigen 1 (Sca1). In addition, the absence of hematopoietic (CD45, CD11c, and CD34) and endothelial markers (CD31) was assessed as previously described^6^. All mAbs were purchased from BD Pharmingen (San Diego). Isotype-matched antibodies were used as controls. For immunophenotypic analysis, ASC were incubated at 4 °C for 10 min with 15% adult bovine serum followed by incubation with the specific mAb at 4 °C for 30 min. At least 10,000 events were analyzed by flow cytometry on a FACScalibur (Becton Dickinson) using the Cell Quest software (Becton Dickinson).

### Isolation and characterization of ASC nanovesicles

NVs were isolated from the culture medium of murine ASC at 14–18 passages, as previously described^27^. ASC were grown to confluence and were maintained for 72 hours in DMEM supplemented with 0.5% of ultracentrifuged FBS to reduce the contamination of FBS-derived vesicles. Culture medium (120 ml) from ASC was collected and subjected to two-step centrifugation at 4 °C (80 g for 5 min + 1300 g for 10 min) to remove floating cells and debris. NVs were obtained by filtration using a 0.22 µm filter (Millipore) and a membrane concentrator (MWCO 5 K, Corning Spin-X UF20) at 3200 g for 90 min at 4 °C. The fraction was purified with two ultracentrifugations at 100,000 g (Optima Max-E Ultracentrifuge, Beckman Coulter) for 1 hour at 4 °C. The pellet obtained was re-suspended in buffer containing a protease inhibitors cocktail (Roche). The protein content was quantified with the BCA protein assay kit, following manufacturer’s instructions (Pierce). To confirm NV isolation and purity, 15–20 µg of NVs were eluted with loading buffer, boiled for 5 min and separated in 10% and 12% polyacrylamide gels at 100–150 V. Proteins were transferred onto polyvinylidene difluoride membranes (Immobilon P, Millipore) for 2 hours at 60 V in transfer buffer. Membranes were then blocked with 10% non-fat dry milk for 1 hour and incubated with anti-CD9 mAb (Millipore; 1:500), anti-TSG101 mAb (Abcam; 1:1000) and polyclonal anti-HSP70 (Santa Cruz Biotechnology; 1:1000). After incubation with the appropriate secondary IgG HRP-conjugated antibodies, the membranes were developed with Immobilon^TM^ Western substrate (Millipore) and proteins were visualized on autoradiography film (Hyperfilm, Amersham Biosciences).

### *In vitro* and *in vivo* CD4^+^ T cell proliferation assays

Myelin oligodendrocyte glycoprotein (MOG)-specific CD4^+^ T cells were isolated from lymph nodes and spleens of 2D2-T cell receptor (TCR)-transgenic mice (the Jackson Laboratories^39^) by magnetic cell sorting, following manufacturer’s instructions (Miltenyi Biotech). An amount of 0.3 × 10^6^ CD4^+^ cells were incubated in complete medium (RPMI + 10% FBS + sodium pyruvate 1 mM + penicillin/streptomycin) in 96-well plates with increasing concentration of MOG_35–55_ peptide (GenScript), in the presence of 1.2 × 10^6^ irradiated splenocytes as antigen presenting cells. In some experiments, cells were stimulated in the presence of 6, 15 or 30 ng/ml ASC-NVs. After 2 days of activation, [^3^H]-thymidine (1 µCi/well) was added for 18 hours before the culture termination. [^3^H]-thymidine uptake was determined with a µ-counter (Perkin Elmer) after the addition of Ultima Gold^TM^ scintillation cocktail (Perkin-Elmer) to each sample.

To assess the effect of ASC-NVs on T cell proliferation *in vivo*, C57Bl/6 mice were immunized for EAE induction as described below. PBS (control mice) or 4 μg of ASC-NVs were injected in immunized mice 3 and 8 days dpi. 4 hours after the second injection, 15 × 10^6^ total cells from lymph node and spleen of naïve 2D2-TCR mice were labeled with green carboxyfluorescein succinimidile ester (CFSE; eBioscience) and infused in immunized mice. Three days later (11 dpi), recipient mice were perfused with PBS and draining lymph nodes were collected and mechanically dissociated. Cells were labeled with allophycocyanin (APC)-conjugated rat anti-mouse CD4 antibody (Biolegend) and the proliferation of CD4^+^CFSE^+^ T cells was determined by flow cytometry analysis with FlowJo software (Tree Star Inc.), as previously described^40^.

### *In vitro* proliferation assay for microglial N9 cell line

The proliferation of microglial N9 cells was assessed by commercial BrdU proliferation Kit (Cell proliferation Elisa, BrdU; Roche Diagnostic Gmbg) according to the manufacturer’s instructions. Briefly, N9 cells were seeded into a 24-well plate and stimulated with 100 ng/ml of lypopolysaccharide (LPS) for 24 h and then treated with 15 ng/ml and 30 ng/ml of NV for further 48 h. Cells were then incubated with BrdU solution at 37 °C. Absorbance was measured at 450 nm with a spectrophotometer (BioRad Laboratories). The rate of mitotic activity was proportional to fluorescence emission. Data are presented as fluorescence arbitrary units (a.u.) relative to the basal (not stimulated) condition.

### MultiPlex assay for cytokines production

Supernatants from proliferation assays in which cells were treated with 30 ng/ml NVs were used to evaluate cytokine production by proliferating T cells. A Multiplex cytokine assay (MilliPlex, Millipore) was performed, following manufacturer’s instructions. The following cytokines were detected and quantified in the assay: interleukin (IL)-1β, IL-1α, IL-4, IL-5, IL-6, IL-10, IL-17, interferon (IFN)-γ, granulocyte macrophage colony-stimulating factor (GM-CSF) and tumor necrosis factor (TNF)-α.

### Preparation of MOG-specific activated T cells

Total splenocytes from 2D2-TCR transgenic mice were stimulated *in vitro* with 30 µg/ml MOG_35–55_ peptide in complete medium (10 × 10^6^ cells/ml). After 4 days of stimulation, cells were washed, and live cells were isolated by Ficoll-Paque gradient. Cells were then incubated for 24 hours with PBS (control) or 6, 15 or 30 ng/ml ASC-NVs in complete medium. After 24 hours, cells were washed and immediately used for *in vitro* adhesion assays or intravital microscopy experiments.

### *In vitro* adhesion assays on purified integrin ligands

Glass slides were coated overnight at 4 °C with 1 µg/ml purified mouse intercellular adhesion molecule (ICAM)-1 or vascular cell adhesion molecule (VCAM)-1 (R&D Systems) in PBS. MOG-specific T cells were treated with NVs as described above and left to spontaneously adhere on ICAM-1 or VCAM-1 for 20 min at 37 °C. In some experiments, chemokine CXCL12 (SDF-1; 0.5 µM) was added for 5 min to evaluate the chemokine-induced adhesion. Cells were then immediately fixed in glutaraldehyde 1.5% in ice cold PBS and counted by computer-assisted enumeration^41^.

### Flow cytometry

MOG-specific T cells were incubated for 24 hours with 30 ng/ml ASC-NVs. The following rat-anti mouse purified antibodies were used to detect adhesion molecule by antigen-specific splenocytes: anti-lymphocyte function-associated antigen-1 (LFA-1; TIB-213 clone), anti-α_4_-integrin (PS/2 clone), anti-P-selectin glycoprotein ligand-1 (PSGL-1; 4RA10 clone) and anti-CD44 (MJ64 clone). Adhesion molecule expression was then quantified by flow cytometry after staining with phycoerythrin (PE)-conjugated secondary goat anti-rat antibody (Biolegend).

### EAE induction and treatment protocol

Chronic EAE was induced in 6–8 weeks old C57Bl/6 mice by subcutaneous immunization with 300 μg of MOG_33–35_ peptide in complete Freud’s adjuvant (Becton Dickinson) containing 0.8 mg/ml *Mycobacterium Tuberculosis* (Becton Dickinson), as previously described^42,43^. Mice were also injected intravenously (i.v.) with pertussis toxin (40 ng; List Biological Laboratories) at the day of immunization and after 48 hours. To evaluate the clinical and pathological efficacy of ASC-NVs in chronic EAE, mice were injected i.v. with NVs at 3, 8 and 13 dpi (preventive protocol) or at 12, 16 and 20 dpi (therapeutic protocol). For each injection, we infused 5 μg of NVs in 0.3 ml of PBS. Control mice received only vehicle. Clinical score was blindly registered according to the following scale: 0 = healthy, 1 = limp tail, 2 = ataxia and/or paresis of hindlimbs, 3 = paraplegia, 4 = paraplegia with forelimb weakness or paralysis, 5 = moribund or death animal. Animals were housed in pathogen-free, climate-controlled facilities and were provided with food and water according to current European Community laws. All mouse experiments were carried out in accordance with experimental guidelines approved by the University of Verona committee on animal research (Centro Interdipartimentale di Servizio alla Ricerca Sperimentale) and by the Italian Ministry of Health.

### Histology and immunohistochemistry

Frozen sections were obtained from lumbar and dorsal spinal cord and processed for histology and immunohistochemistry at 25 dpi, as previously described (11). Histological assessment of spinal cord demyelination (Woelcke staining) and inflammatory infiltrates (hematoxylin and eosin; H&E) in lumbo-sacral segments was blindly performed calculating affected areas in at least three sets (100 µm apart) of six sections each for every single animal. For immunohistochemistry, anti-CD3 (for T cells; Serotec, Oxford, UK) and anti-Iba1 (for monocytes/macrophages; Wako) primary antibodies were incubated overnight. The numerical density of microglia and the area of microglial cells were determined in 10 μm spinal cord sections. Images were acquired from spinal cord using a LEICA microscope at a magnification of 40× and blindly counted with ImageJ v1.32j software.

To evaluate the expression of cell adhesion molecules, a heat-induced epitope retrieval was required with epitope retrieval buffer, pH 8.0 (Leica) at 90 °C. Sections were then treated with 20% normal goat serum (Vector Laboratories, Burlingame, CA) and 1% BSA and then incubated with primary antibodies over night at 4 °C. We used mAbs to VCAM-1 (1:500, GTX82989 clone, Genetex), P-Selectin (1:10 ab6632 clone, Abcam), ICAM-1 (1:100, ab2213 clone, Abcam) and E-Selectin (1:20, BBA16 clone, R&D). After washing, appropriated biotinylated secondary antibodies were added; the reaction was visualized with ABC kit (Vector) and Novared Substrate Kit (Vector) according to manufacturer instructions. Images of immune-peroxidase were obtained with Zeiss Axiophot microscope equipped with Axiocam HCR camera and Axiovision software. Lesions were identified on digital images and determined following a manual outline of the lesion border and expressed as percentage of the total spinal cord section area. All counts were performed in three sets for each animal.

### Intravital microscopy experiments in spinal cord venules

MOG_35–55_-immunized EAE mice at disease peak were anesthetized by intraperitoneal injection of ketamine (100 mg/kg body weight)/xylazine (15 mg/kg) solution. After skin removal, a spinal column stabilization device was made, by mounting STS-A Compact Spinal Cord Clamps and MA-6N head holding adaptor on a steel base that was adapted to fit on the microscope’s stage. These instruments (Narishige, Japan) were adapted on a customized microscope stage (Siskiyou, USA) in our intravital microscopy facility. A midline dorsal incision exposed the lumbar column over L1-L4, the main site of inflammation in EAE. Muscles were dissected from the sides of the vertebral bone and laminectomy was performed with micro drill and bone scraper to expose the spinal cord. We left an intact dura mater (approximately 50 µm) to protect the spinal cord, and further dampened the spinal cord motions by sealing with a drop of low melt agarose on the dura and a coverglass. Dorsal spinal cord vessels were visualized by i.v. injection of fluorescent dextran (Molecular Probes). MOG_35–55_-specific T cells were incubated for 24 hours with PBS or 30 ng/ml ASC-NVs, and then labeled with 5-(6)-(((4-Chloromethyl)Benzoyl)Amino)Tetramethylrhodamine (CMTMR) for 20 min at 37 °C or with 40 mM 5-Chloromethylfluorescein Diacetate (CMFDA) (Molecular Probes, USA) for 4 min at 37 °C. 30 × 10^6^ cells were injected i.v. in recipient mice immediately before the acquisition. We measured the number of rolling interactions, rolling velocity, hemodynamic parameters and cell sticking, defined as firm adhesion for at least 10 sec. Analysis of intravital microscopy experiments was performed as previously described^42,44^.

### Statistical analysis

Data are presented as mean ± standard deviation (SD) or standard error of the mean (SEM). Statistical analysis using a two-tailed Student’s *t*-test was performed to evaluate differences between ASC-NVs and control treatments for several parameters. A *p value* < 0.05 was considered statistically significant (*p < 0.05; **p < 0.01; ***p < 0.001; ****p < 0.00001).
